# A Novel miRNA Detection Method Using Loop-Mediated Isothermal Amplification

**DOI:** 10.1155/2023/6624884

**Published:** 2023-09-12

**Authors:** Saiwei Wu, Abdu Ahmed Abdullah Al-Maskri, Qun Li, Jiatong Liu, Sheng Cai

**Affiliations:** ^1^Department of Pharmacy, The Fourth Affiliated Hospital, Zhejiang University School of Medicine, Yiwu, Zhejiang, China; ^2^Institute of Drug Metabolism and Pharmaceutical Analysis, College of Pharmaceutical Sciences, Zhejiang University, Hangzhou, Zhejiang 310058, China

## Abstract

A novel ligation-based loop-mediated isothermal amplification has been developed for miRNA detection. Two stem-loop structure DNA linker A/B probes which hybridized with miRNA were designed to establish a rapid and ultrasensitive miRNA-LAMP system for miRNA detection. Target miR-200a was used to template the ligation of Linker A/B probes with SplintR Ligase and used as a dumbbell-shaped amplicon. By adding BIP/FIP and Bst 2.0 DNA polymerase, the LAMP reaction was carried out, which brought greatly improved amplification efficiency. The double-stranded DNA fluorescent dye EvaGreen was added for the detection of amplification product to achieve the quantification of the target miRNA. This method can detect miRNA in a linear range of seven orders of magnitude, with a detection limit of 100 fM. Therefore, this ultrasensitive miRNA-LAMP assay provides a new path for the highly sensitive quantitative analysis of miRNA, thereby bringing convenience to clinical diagnosis and prognostic research.

## 1. Introduction

MicroRNAs (miRNAs) comprise a family of small single-stranded non-protein-coding RNAs (20–25 nucleotides) that have recently emerged as post-transcriptional regulators of gene expression [1, 2]. In addition, miRNAs play critical roles in various biological processes and genetic pathways. They are also involved in a number of human diseases including cancer [3–6], which have been hypothesized to play an important role in the control of tumor growth and eventually lead to more roles and progression [7]. miRNAs have been regarded as promising biomarkers for clinical diagnosis and treatment [8, 9]. However, miRNA detection is very challenging because of the intrinsic characteristics of these molecules, such as small size, sequence similarity among various members, and lack of common features.

Currently, more than 2000 miRNAs have been annotated in humans (miRBase: http://www.mirbase.org/index.shtml), and computational predictions indicate that miRNAs may regulate the expression of 60% of all human protein-coding genes [10]. MiRNAs play a pivotal role in critical biological processes, including cellular growth, proliferation, and differentiation [11, 12]. A growing body of evidence has demonstrated the importance of miRNAs in managing chemotherapy efficacy in multiple human cancers, and they might also function as tumor suppressors and oncogenes. miRNAs have opened a new window to an important area of biology that was previously unexplored and also have important implications in human development and diseases. Thus, the rapid and sensitive quantification of miRNAs is of great importance to the understanding of their biological functions and clinical applications.

At present, the most conventional methods used for detecting miRNA include northern blot analysis [13, 14], stem-loop quantitative reverse transcription polymerase chain reaction (RT-qPCR) [15–17], and microarrays [18, 19]. However, these methods are limited by inaccurate quantification, slow and complicated operation, time-consuming process, and high instrument requirements. Recently, several isothermal amplification tests, e.g., primer extension [20], isothermal exponential amplification techniques such as strand displacement amplification [21–24], rolling circle amplification (RCA) [25–27], nucleic acid sequence-based amplification [28], helicase-dependent amplification [29], recombinase polymerase amplification [30], and loop-mediated isothermal amplification (LAMP) [31–34] have been developed. These methods have been successfully tested on several techniques for miRNA detection and are more efficient than the typical PCR. Thus, they can be applied as alternative methods. Moreover, these methods have great potential for on-site, point-of-care, and in situ assay applications. Isothermal amplification techniques eliminate the need for temperature cycling for PCR, enhancing the PCR amplification yield. Among them, LAMP and RCA are the most commonly used isothermal amplification techniques.

LAMP is an isothermal method that was first described by Notomi et al. [35]. In a typical LAMP reaction, there are at least four primers: two inner and two outer primers. These primers were designed to recognize a set of six distinct sequences, displaced by the on the autocycling strand displacement activity DNA polymerase and generates a large amount of amplified products within 1 h; moreover, the method can be simplified by running at a constant temperature, eliminating the need for temperature cycling during amplification [34]. The mechanism of LAMP amplification reaction includes three steps: production of starting material, cycling amplification and elongation, and autocycling strand displacement. Therefore, the limit of detection and dynamic range of the nucleic acid will be highly useful depending on the nature of the bio-fluid tested from patients (e.g., whole blood, serum, or plasma) [36]. In the last decade, most studies determined whether LAMP with biosensor could distinguish between nucleic acid that differs by a single base mismatch.

**Scheme 1:**
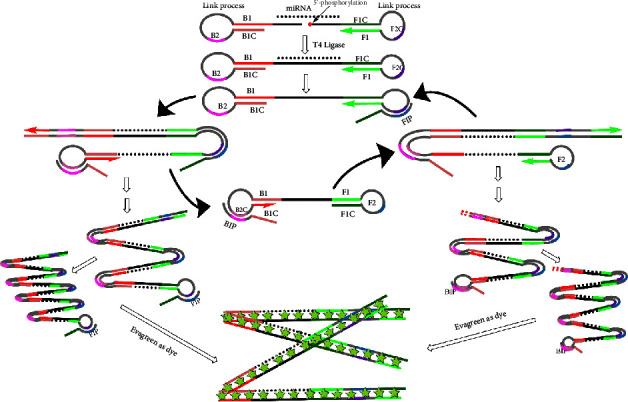
Schematic representation of miRNA detection using the miRNA-LAMP system.

We have previously developed a reverse transcription-based LAMP (RT-LAMP) to provide a highly selective and sensitive platform for miRNA [37]. In this work, we also used the LAMP reaction for miRNA detection, but reverse transcription process was replaced by ligation reaction to make the method simple and sensitive. Two stem-loop structures DNA (Linker A/B probes) were designed, which were hybridized with miRNA and linked by SplintR Ligase when the target miRNA appeared. The LAMP reaction was carried out by adding backward inner primer (BIP)/forward inner primer (FIP) and Bst 2.0 DNA polymerase. Compared with other self-assembly amplification methods, LAMP greatly improved the amplification efficiency. Finally, we added the double-stranded DNA fluorescent dye EvaGreen to detect the amplification product and achieve the quantification of the target miRNA, as shown in Scheme 1. The selectivity of this method was evaluated by testing miR-200a and other miRNAs from the miR-200 family. Our method can effectively distinguish miR-200a from other homologous family miRNAs with high sequence similarity. This method has also been successfully applied to quantitatively detect the amount of miR-200a in cell samples, indicating its clinical value. Therefore, this novel detection method could open a new path for the highly sensitive quantitative analysis of miRNA, thereby bringing convenience to clinical diagnosis and prognostic research.

## 2. Materials and Methods

### 2.1. Material and Apparatus

miRNA 1st Strand cDNA synthesis kit was purchased from Vazyme (Nanjing, China), and Bst 2.0 DNA polymerase and the corresponding isothermal buffer were purchased from New England Biolabs (USA); dNTPs and RNase-free water were all obtained from Takara (Dalian, China). All nucleic acid in this work including Linker A, Linker B, and inner primer (FIP and BIP) were synthesized and purified by Sangon Biotech (Shanghai, China). miRNAs were synthesized and purified by Genepharma (Shanghai, China). The sequences of all nucleic acids are listed in Table S1. The real-time fluorescence measurements of the LAMP reactions were proceeded on a StepOne (Applied Biosystems) real-time PCR instrument and had the following sequences.

### 2.2. Procedure of LAMP Assay

In a typical experiment, 9 *μ*L reaction mixture containing 1 *μ*L target miRNA, 1 *μ*L each Linker A and B probes 1, 25 nM and 1 *μ*L of 0.8 mM dNTPs, 1x mM in SplintR Ligation buffer was heated to 95°C for 5 min and chilled on ice for 2 min, then 1 *μ*L 7 U of SplintR Ligase was added and incubated at 16°C for 30 min. The SplintR mixture solution (10 *μ*L) was added into a total volume of 20 *μ*L in 1x isothermal amplification buffer to form LAMP Mix composed of forward inner primer (FIP), backward inner primer (BIP) 80 nM, and 8 U of Bst 2.0 DNA polymerase. 1 × EvaGreen dye was added to the reactions following primer annealing but prior to the addition. The reactions were analyzed using the Step OnePlus real-time PCR machine that was set up to incubate the samples for 60 cycles of two-step incubations: step 1: incubation at 61.5°C for 150 s and step 2: incubation at 61.5°C for 30 s (total incubation time of 3 min/cycle unless otherwise indicated). The resulting data were analyzed using the Step OnePlus analysis software to generate Ct (quantification cycle) values for each amplification, and Ct was redefined by multiplying Ct by 3 in this paper.

### 2.3. Cell Lysis and RNA Preparation

The human colon cancer cells (HT29) and human hepatoma cells (BEL7402) were gifts from Department of Pharmacology, College of Pharmaceutical Sciences, Zhejiang University. Cell lines were maintained according to instructions from the ATCC. HT29 and BEL7402 were collected and centrifuged at 3000 rpm for 5 min in culture medium, washed once with PBS buffer, and then spun down at 3000 rpm for 5 min. Total RNA was extracted from human colon cancer cells using miRNeasy Kit according to the manufacturer's procedures. The sample of miR-200a in these cells was diluted and then analyzed with the proposed miRNA detection method.

## 3. Results and Discussion

### 3.1. Design Principle of this Method Based LAMP

This miRNA detection strategy contains two linker probes and two primers; both of them are from the typical LAMP reaction. The LAMP system, which consists of two hairpin DNA probes (Linker A and Linker B) are designed and constructed as a dumbbell DNA initiator to get an active amplicon. The Linker A/B probes have an overhang complementary to the half of target miRNA. Only when it is perfectly hybridized with miRNA, the ligation of these two probes would be carried out in the presence of SplintR Ligase to afford high specificity in identifying the mutations in target miRNA. The LAMP process would start with the coming of FIP/BIP primer where intact inner primers bind and extend. The fluorescent dye (1x EvaGreen) was added to the reactions following primer annealing with the addition of Bst 2.0 DNA polymerase. In the absence of miRNA or there is a mismatch between miRNA, no dumbbell-shaped amplicons structured product is obtained, therefore, precluding the LAMP amplification with merely giving a low and a late increase of the fluorescence signal. This miRNA-LAMP assay could get a high sensitivity and selectivity according to the real-time fluorescence curve and Ct value which were recorded in LAMP reaction.

### 3.2. Design of Linker A/B Probes

**Figure 1:**
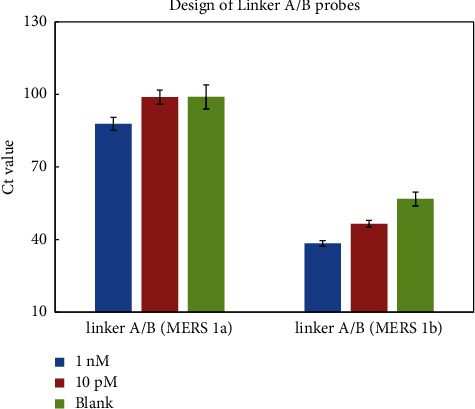
Comparison of Linker A/B (MERS 1a and MERS 1b). Experimental conditions: Linker A/B 10 pM, 0.8 mM dNTP, 4 U SplintR Ligase, 46 nM FIP and BIP primers, and 8 U Bst 2.0 DNA polymerase. Detection was performed as described in material and methods.

To explore the feasibility and more investigate of this method in practical use, we designed and compared two templates (MERS 1a and MERS 1b), which came from the original LAMP. This two Linker A/B probes extension mechanism of the miRNA-LAMP system were tested for miR-200a detection, which is well a known biomarker overexpressed in the luminal breast cancer (strongly downregulated during oncogenic EMT) [42]. The switch from loop structure is a key factor for the amplification mechanism, thus the design of Linker A/B probes is important for the detection. The real-time fluorescence intensity distribution curves were used to investigate the efficiency of Linker A/B probes from two templates. After we tested in parallel with different concentrations from 1 nM and 10 pM of target miR-200a, the probes from MERS 1b template showed high sensitivity and changed in the fluorescence intensity function of target concentration by real-time RT-PCR, as shown in (Figure 1).

### 3.3. Amplification Reaction Steps for Detection Method

**Figure 2:**
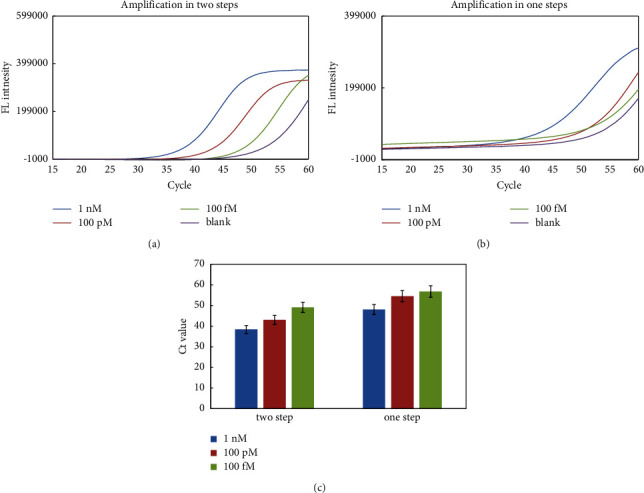
Comparison of amplification reaction steps for miRNA-LAMP assay. (a and b) Real-time fluorescence intensity, (c) *Ct* value for one or two amplification reaction steps methods. Experimental conditions: Linker A/B 10 pM, 0.8 mM dNTP, 4 U SplintR Ligase, 46 nM FIP and BIP primers, and 8 U Bst 2.0 DNA polymerase. Detection was performed as described in material and methods.

To achieve a highly sensitive detection of miRNA, we optimized the miRNA-LAMP reaction by investigating different operation strategies. One-step reaction method where we put all the enzyme, target, and primers in one tube and then start the amplification directly were compared with two-step method (ligation and amplification). The quality of the Ct value for different concentrations of the target are shown in Figures 2(a)–2(c); the two-step method showed better performance in the amplification curve and enhanced sensitivity for miRNA detection.

### 3.4. Ligase Enzyme Selection

**Figure 3:**
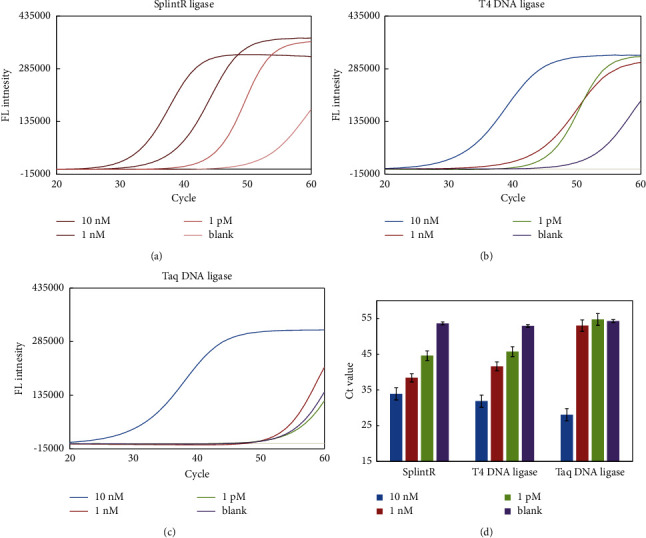
Comparison of different ligase enzymes. (a) SplintR Ligase, (b) T4 DNA Ligase, and (c) Taq DNA ligase were analyzed for the real-time fluorescence intensity, (d) *Ct* value for three enzymes. Experimental conditions: Linker A/B 10 pM, 0.8 mM dNTP, 4 U SplintR Ligase, 46 nM FIP and BIP primers, and 8 U Bst 2.0 DNA polymerase. Detection was performed as described in material and methods.

To achieve the highest-efficiency ligation between the Linker A/B probes mediated by ligase enzyme to form the dumbbell-shaped, the approach was optimized by testing three different types of ligase enzymes. SplintR Ligase, Taq DNA Ligase, and T4 DNA Ligase were applied miRNA-LAMP detection. As shown in the (Figures 3(a)–3(d)), results showed that the SplintR Ligase demonstrated high efficiency compared with the other two enzymes (T4 DNA Ligase and Taq DNA Ligase), which ligated the two adjacent single-stranded DNA splinted fragments with high efficiency by a complementary miRNA strand.

### 3.5. Optimization for the miRNA-LAMP Assay

To establish the detection method and reduce the number of false-positive results, several parameters were systematically investigated for miRNA-LAMP detection. These parameters included the concentrations of Linker A/B, FIP and BIP primers, SplintR Ligase, Bst 2.0 DNA polymerase, and the reaction temperature. The concentration of Linker A/B led to an increase in the Δ*Ct* value, which reached the maximum at 50 nM and then decreased gradually (Figure S1). The Δ*Ct* value reached the maximum when primers FIP/BIP concentration is 800 nM and then decreased gradually (Figure S2). The Δ*Ct* value also reached a maximum for a Bst 2.0 DNA polymerase amount of 8 U (Figure S3) and SplintR Ligase amount of 4 U (Figure S4). Furthermore, we investigate the LAMP amplification temperature ranging from 59°C to 65°C; the Δ*Ct* values for each different amplification temperature were compared when the amplification temperature is 61.5°C and the results show lower Δ*Ct* values from miRNA (Figure S5).

### 3.6. Selectivity

**Figure 4:**
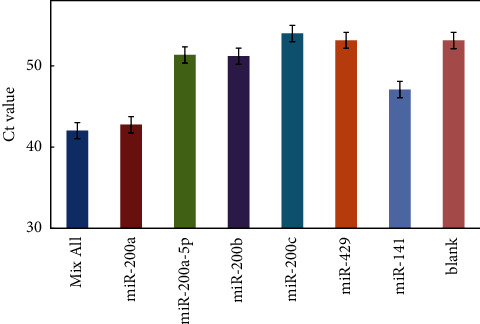
The selectivity of the analysis of miR-200a/mismatch miRNA. Experimental conditions: Linker A/B 10 pM, 0.8 mM dNTP, 4 U SplintR Ligase, 46 nM FIP and BIP primers, 8 U Bst 2.0 DNA polymerase. Detection was performed as described in material and methods.

To evaluate the selectivity of this miRNA-LAMP method, interference assays were performed under identical conditions using other miRNA from miR-200 family, such as miR-200b, miR-200c, and miR-429, due to the high sequence homology (Table S1). As shown in Figure 4, the *Ct* value was measured from perfectly complementary targets miR-200a (1 pM) to other miR-200 family miRNAs (10 pM). The results suggested that high amplification and lowest Ct value for miR-200a target compared to other members of the family, which demonstrated that this approach for detection miRNA had high selectivity and sensitivity. Our miRNA assay can effectively discriminate between members of multiple closely related sequences of miRNAs from the miR-200 family.

### 3.7. Quantification of miRNA-LAMP Assay

**Figure 5:**
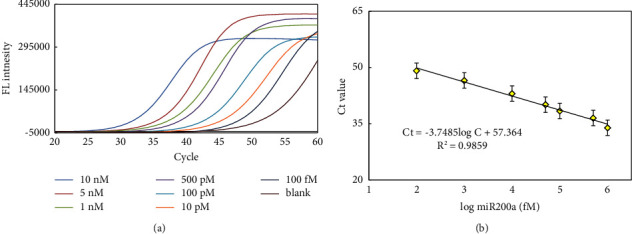
Fluorescence intensities (a) and *Ct* values (b) of miRNA-LAMP assay. Experimental conditions: Linker A/B 10 pM, 0.8 mM dNTP, 4 U SplintR Ligase, 46 nM FIP and BIP primers, and 8 U Bst 2.0 DNA polymerase. Detection was performed as described in material and methods.

Under the optimized conditions, different concentrations of miR-200a were analyzed by monitoring the changes in the *Ct* value. The real-time fluorescence intensity curves all exhibited a sigmoidal shape, and gradual increases of the *Ct* values were observed with decreased miR-200a concentrations from 100 fM to 10 nM. A fluorescence signal produced by a miR-200a concentration as low as 100 fM could be clearly discriminated from that of the blank control (Figure 5). The miR-200a ranged between 100 fM to 5 nM, with the correlation equation *Ct* = −3.7485l og C + 57.364 (*R*^2^ = 0.9859). The sensitivity of this assay compared favorably with previous efforts for miRNA detection, as summarized in (Table S2).

### 3.8. Assay of miRNA in Cell Samples

The proposed methods were successfully used to quantify the amount of miR-200a in the total RNA sample that was extracted from cells. Human colorectal cancer cells HT29 were used as positive control and human liver cancer cell BEL-7402 as a negative control (low expression miR-200a). We detected the miR-200a level from the total RNA extracted concentration not exceeding 250 ng; the results of miRNA-LAMP assay were in good agreement with those obtained from other stem-loop RT-PCR. miR-200a in two types of cells HT29 and BEL-7402 were detected. The results indicate that HT29 and BEL-7402 cells contain 73.3 pM and 6 fM of miR-200a in total RNA 415 ng and 316.8 ng.

## 4. Conclusion

In this section, a simple and efficient miRNA-LAMP detection method based on development self-assembly amplification to LAMP is established. In this method, the smart design of the two probes with the loop was specifically designed to form two different stem-loop structures before and after binding to the target miRNA. The sensitivity of the LAMP method is comparable to that of the self-assembly amplification method, but the upper limit of quantification of the miRNA-LAMP method is higher and showing a wider range of quantification, which is feasible for the accurate detection of miRNAs down to the 100 fM level in real samples. Moreover, this proposed miRNA-LAMP assay does not require any modified or labeled DNA probes, and only one type of ligase enzyme with DNA polymerase is needed, which should significantly reduce the cost and simplify the experimental procedure. At the same time, the miRNA-LAMP-based miRNA detection method we just need to modify the complementary sequences of the probe and target miRNA can easily be extended to the detection of other small RNAs, including miRNA, so it has universal value. The versatile miRNA-LAMP miRNA assay not only remarkably simplifies the probe design for efficient LAMP amplification but also leads to high sensitivity and specificity.

## Data Availability

The data used to support the findings of this study are available from the corresponding author upon request.

## Supplementary Materials

Supplementary Materials Table S1: miRNA and DNA sequences used in this work. Table S2: Comparison of sensitivity for miRNA assay methods, Figure S1: Δ*Ct* value vs. the mount of Linker A/B probes, Figure S2: Δ*Ct* value vs. the amount of FIP/BIP Primer, Figure S3: Δ*Ct* value vs. the amount of Bst 2.0 DNA polymerase, Figure S4: Δ*Ct* value vs. the mount of of SplintR Ligase, Figure S5: Δ*Ct* value vs. the different incubation temperatures.
