# Supplementary material for: A Novel miRNA Detection Method Using Loop-Mediated Isothermal Amplification
Source: Int J Anal Chem. 2023 Sep 12;2023:6624884. doi: 10.1155/2023/6624884 (PMC10508998; doi:10.1155/2023/6624884)
Supplement: Supplementary Materials — Table S1: miRNA and DNA sequences used in this work. Table S2: Comparison of sensitivity for miRNA assay methods, Figure S1: ΔCt value vs. the mount of Linker A/B probes, Figure S2: ΔCt value vs. the amount of FIP/BIP Primer, Figure S3: ΔCt value vs. the amount of Bst 2.0 DNA polymerase, Figure S4: ΔCt value vs. the mount of of SplintR Ligase, Figure S5: ΔCt value vs. the different incubation temperatures. [file 6624884.f1.docx]

Supporting information

A Novel miRNA Detection Method using Loop-Mediated Isothermal Amplification

Saiwei Wu ^1^, Abdu Ahmed Abdullah Al-Maskri ^2^, Qun Li ^1^, Jiatong Liu ^2^ and Sheng Cai ^2,^*

^1^ Department of Pharmacy, The Fourth Affiliated Hospital, Zhejiang University School of Medicine, Yiwu, Zhejiang, China;

^2^ Institute of Drug Metabolism and Pharmaceutical Analysis, College of Pharmaceutical Sciences, Zhejiang University, Hangzhou, Zhejiang 310058, China;

* Correspondence should be addressed to Sheng Cai; caisheng@zju.edu.cn

Table S1. MiRNA and DNA sequences used in this work.

| Type | Name | Sequence (5′-3′) |
| --- | --- | --- |
| Target | miR-200a-3p | UAACACUGUCUGGUAACGAUGU |
|  | miR-200a-5p | CAUCUUACCGGACAGUGCUGGA |
|  | miR-200b | UAAUACUGCCUGGUAAUGAUGA |
|  | miR-200c | UAAUACUGCCGGGUAAUGAUGGA |
|  | miR-429 | UAAUACUGUCUGGUAAAACCGU |
| MERS 1b FIP Start | FIP | AGCACCCTCAACATCGAAGCACTCGTGAAGAGGCTGTA |
|  | BIP | TGCTTCCCGTAATGCATGTGGACTGGCTGAACAACAAAGT |
|  | Linker A (FIP) | AGCACCCTCAACATCGAAGCACTCGTGAAGAGGCTGTAAGGCAAGTTCGAAGCTGGATAGGCTTCGATGTTGAGGGTGCTACATCGTTACC |
|  | Linker B (FIP) | AGACAGTGTTATGCTTCCCGTAATGCATGTGGCACCAATGTGCCTCTACAATTAGGATTTTCAACTGGTGTGAACTTTGTTGTTCAGCCAGTCCACATGCATTACGGGAAGCA |
| MERS 1a FIP Start | Linker A (FIP) | AAGCATTAGTGGGGGCAAGCCCCACTACTCCCATTTCGTCAGCGCTGATTGCAGTTGCAAATTGGCTTGCCCCCACTAATGCTTACATCGTTACC |
|  | Linker B (FIP) | AGACAGTGTTAATGCGCACTACACATACTGATATTGGTGTCTACATTAGTATGTCACTTGTATTAGTCATTGTAGTGAAGAGATTGTACAAATATCAGTATGTGTAGTGCGCAT |

Table S2. Comparison of sensitivity for miRNA assay methods.

| Analytical method | Target RNA | Detection Limit | References |
| --- | --- | --- | --- |
| SERS | miR-200a | 120 fM | [1] |
| electrochemical genosensor | miR-200a | 8.4 fM | [2] |
| electrochemical genosensor | miR-200a | 29 fM | [3] |
| SERS | miR-200b | 120 fM | [4] |
| Impedimetric sensor | miR-200c | 1.13 fM | [5] |
| Intelligent dual-drive DNA nanosensor | miR-200c | 5.5 fM | [6] |
| Combination of DNA-peptide Probes and LC-MS/MS | miR-200c | 1 pM | [7] |
| LAMP | miR-200a | 100 fM | This work |

**Optimal conditions for the restriction miRNA-LAMP assay**


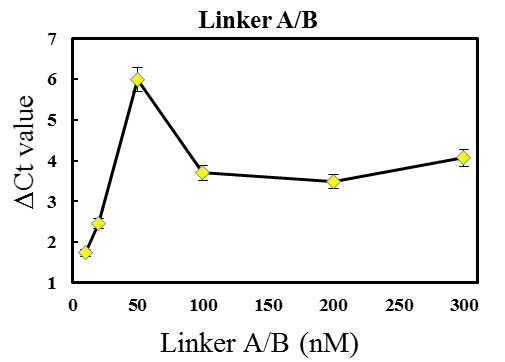


FIGURE S1: ΔCt value vs. the mount of linker A/B probes. Experimental conditions: the total volume of every reaction system was 25 μL, dNTP 0.8 mM, target miR-200a 1 pM, SplintR Ligase 7.1 U, FIP and BIP primers 46 nM, and Bst 2.0 DNA polymerase 8 U. Detection was performed as described in the Experimental section (ΔCt value = Ct value of blank - Ct value of target).


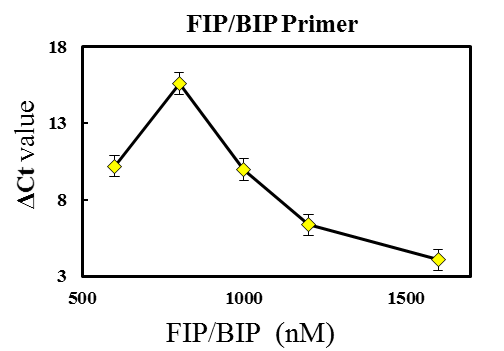


FIGURE S2: ΔCt value vs. the amount of FIP/BIP Primer. Experimental conditions: the total volume of every reaction system was 25 μL, linker A/B 10 pM, dNTP 0.8 mM, target miR-200a 1 pM, SplintR Ligase 7.1 U, and Bst 2.0 DNA polymerase 8 U. Detection was performed as described in the Experimental section (ΔCt value = Ct value of blank - Ct value of target).


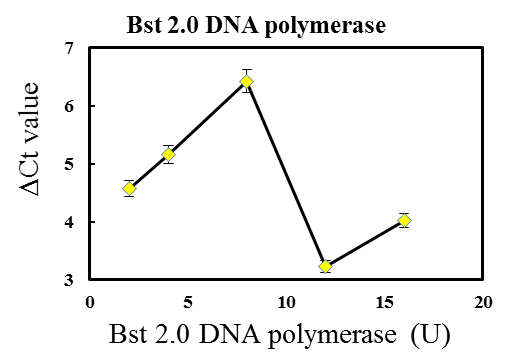


FIGURE S3: ΔCt value vs. the amount of Bst 2.0 DNA polymerase. Experimental conditions: the total volume of every reaction system was 25 μL, linker A/B 10 pM, dNTP 0.8 mM, target miR-200a 1 pM, SplintR Ligase 7.1 U, and FIP and BIP primers 46 nM. Detection was performed as described in the Experimental section (ΔCt value = Ct value of blank - Ct value of target).

**
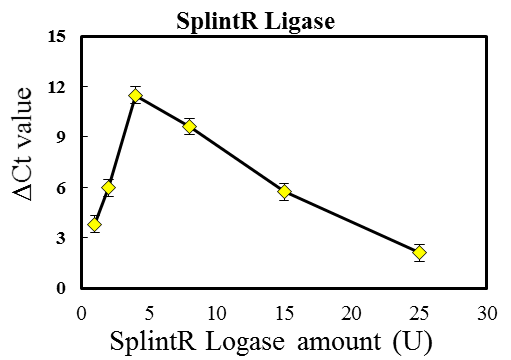
**

FIGURE S4: ΔCt value vs. the amount of SplintR Ligase. Experimental conditions: the total volume of every reaction system was 25 μL, linker A/B 10 pM, dNTP 0.8 mM, target miR-200a 1 pM, FIP and BIP primers 46 nM, and Bst 2.0 DNA polymerase 8 U. Detection was performed as described in the Experimental section (ΔCt value = Ct value of blank - Ct value of target).


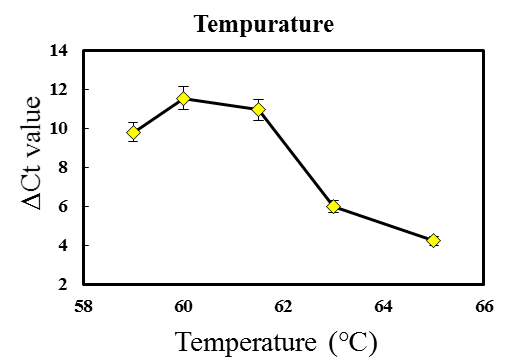


FIGURE S5: ΔCt value vs. the different incubation temperatures. Experimental conditions: the total volume of every reaction system was 25 μL, linker A/B 10 pM, dNTP 0.8 mM, target miR-200a 1 pM, SplintR Ligase 7.1 U, FIP and BIP primers 46 nM, and Bst 2.0 DNA polymerase 8 U. Detection was performed as described in the Experimental section (ΔCt value = Ct value of blank - Ct value of target).

**Reference**

[1] H. Zhang, C. Fu, S. Wu, Y. Shen, C. Zhou, J. Neng, Y. Yi, Y. Jin, Y. Zhu, "Magnetic-Capture-Based Sers Detection of Multiple Serum Microrna Biomarkers for Cancer Diagnosis", *Analytical Methods*, vol. 11, no. 6, pp. 783-93, 2019.

[2] M. Moazampour, H.R. Zare, Z. Shekari, "Femtomolar Determination of an Ovarian Cancer Biomarker (Mir-200a) in Blood Plasma Using a Label Free Electrochemical Biosensor Based on L-Cysteine Functionalized Zns Quantum Dots", *Analytical Methods*, vol. 13, no. 17, pp. 2021-29, 2021.

[3] M. Moazampour, H.R. Zare, Z. Shekari, S.M. Moshtaghioun, "Development of an Electrochemical Genosensor for Quantitative Determination of Mir-200a Based on the Current Response of Ferrocene-Functionalized Graphene Oxide Nanosheets", *Microchemical Journal*, vol. 185, pp., 2023.

[4] H. Zhang, C.P. Fu, S.T. Wu, Y.Q. Shen, C.H. Zhou, J. Neng, Y. Yi, Y.C. Jin, Y.F. Zhu, "Magnetic-Capture-Based Sers Detection of Multiple Serum Microrna Biomarkers for Cancer Diagnosis", *Analytical Methods*, vol. 11, no. 6, pp. 783-93, 2019.

[5] O.A. Vural, Y.T. Yaman, G. Bolat, S. Abaci, "Human Serum Albumin-Gold Nanoparticle Based Impedimetric Sensor for Sensitive Detection of Mirna-200c", *Electroanalysis*, vol. 33, no. 4, pp. 925-35, 2021.

[6] Z.G. Tang, F. Sun, Y.Q. Chai, C.S. Zhang, J. Wang, H.X. Li, "Intelligent Dual-Drive DNA Nanosensor for Ultrasensitive Detection of Prostate Cancer-Related Circulating Microrna-200c", *Microchemical Journal*, vol. 192, pp., 2023.

[7] F.F. Xu, W.X. Zhou, J.X. Cao, Q.Q. Xu, D.C. Jiang, Y. Chen, "A Combination of DNA-Peptide Probes and Liquid Chromatography-Tandem Mass Spectrometry (Lc-Ms/Ms): A Quasi-Targeted Proteomics Approach for Multiplexed Microrna Quantification", *Theranostics*, vol. 7, no. 11, pp. 2849-62, 2017.
